# Population-Attributable Risk Estimates for *Campylobacter* Infection, Australia

**DOI:** 10.3201/eid1505.081553

**Published:** 2009-05

**Authors:** Iain Gillespie

**Affiliations:** Health Protection Agency Centre for Infections, London, UK

**Keywords:** Enteric infections, bacteria, Campylobacter infection, population-attributable risk, chicken, foodborne, Australia, letter

## To the Editor

Many industrialized countries have a high incidence of *Campylobacter* infections. An estimated 250,000 cases of *Campylobacter* infection occur annually in the United States (*1*), and several sequelae compound the impact of these infections. The incidence of *Campylobacter* infections is also important to policy-makers—in the United Kingdom it is used to assess foodborne disease–reduction strategies (*2*)—and governments worldwide rely on the findings of epidemiologic and microbiological studies on *Campylobacter* infection to shape their food-safety policies.

Population-attributable fractions provide added value in case–control studies by helping researchers identify the most important risk factors for a condition on the basis of risk association and frequency of exposure. In an analysis of data from a previous case–control study of *Campylobacter* infection (*3*), Stafford et al. (*4*) used population-attributable fractions to estimate the annual number of *Campylobacter* infection cases among Australians >5 years of age that were attributable to each risk factor from that study. Using this technique, they estimated that 50,500 cases annually can be attributed directly to eating chicken.

Population-attributable fractions have been defined as “the proportion of disease cases over a specified time that would be prevented following elimination of … exposure [to the specified risk factors]” (*5*). Therefore, removing exposure to factors not associated with disease risk will not affect disease incidence. Stafford and colleagues implicitly acknowledge this in their methods: “We calculated PARs [population-attributable risks] … for each variable that was significantly associated with an increased risk for infection.” It is surprising, therefore, that they subsequently included consumption of cooked chicken in their extrapolation, even though this exposure was not significantly associated with illness (adjusted odds ratio 1.4, 95% confidence interval 1.0–1.9, p = 0.06). Because they attributed 35,500 of the 50,500 cases of *Campylobacter* infection to the consumption of cooked chicken, I believe that Stafford et al. overestimated the role of chicken consumption in cases of *Campylobacter* infection by a factor of 3.4.
