# Supplementary material for: Population-Attributable Risk Estimates for Campylobacter Infection, Australia
Source: Emerg Infect Dis. 2009 May;15(5):850–2. doi: 10.3201/eid1505.081553 (PMC2687014; doi:10.3201/eid1505.081553)
Supplement: Related texts

# Reply: In Response

**Authors:** Russell J. Stafford, Philip J. Schluter, Martyn D. Kirk, Andrew J. Wilson

**Affiliations:** Queensland Health, Brisbane, Queensland, Australia (R.J. Stafford, A.J. Wilson); University of Queensland, Brisbane (R.J. Stafford, P.J. Schluter, A.J. Wilson); Auckland University of Technology, Auckland, New Zealand (P.J. Schluter); OzFoodNet, Canberra, Australian Capital Territory, Australia (M.D. Kirk)

Gillespie (*1*) questions whether we should have treated consumption of cooked chicken as a contributing factor in estimating population-attributable risk (PAR) for *Campylobacter* infection because it was not a significant risk factor (adjusted odds ratio [aOR] 1.4, 95% confidence interval [95% CI] 1.0–1.9, p = 0.06). Although on strict statistical grounds Gillespie is correct, we believe that consumption of cooked chicken warrants consideration as a risk factor on the basis of biological plausibility, possible misclassification of the “cooked” status of chicken eaten by study participants, and previous empirical evidence.

Many case–control studies have demonstrated that consumption of any chicken is a risk factor for *Campylobacter* infection (*2*,*3*), although others have shown that only the consumption of undercooked chicken or chicken eaten outside of the home is a risk factor (*4*,*5*). We acknowledged in our study that some misclassification of exposure is likely in any study of reported food-consumption habits and that such misclassification is a major limitation of case–control studies. Because of the difficulty of determining whether previously eaten chicken had been thoroughly cooked or recontaminated after having been cooked, particularly if it was purchased outside of the home, at least some study participants who reported eating cooked chicken could have acquired their infection from the chicken meat. Moreover, as noted above, results from other case–control studies showing an association between disease risk and consumption of chicken or poultry have been based on study participants’ reported consumption of both undercooked and cooked chicken.

In our study, we included chicken consumption in our multivariable models as either “cooked chicken” or “undercooked chicken” but not as “chicken.” However, in our univariate analysis, consumption of any chicken (i.e., cooked or undercooked) was significantly associated with illness (OR 1.6, 95% CI 1.2–2.1), and it was also significantly associated with illness when included in a multivariable model as a single variable (aOR 1.7, 95% CI 1.2–2.3). Results of a univariate assessment of various types of cooked chicken meat showed that consumption of chicken fillet (OR 1.2), chicken kebabs (OR 1.7), and bought barbecued chicken (OR 1.2) was each associated with increased risk for illness. Other studies have similarly shown consumption of cooked, fried, or barbecued chicken to be significantly associated with risk for *Campylobacter* infection (*3*,*4*).

The strength of our approach is that we were able to estimate the number of cases of campylobacteriosis attributable to chicken consumption each year and to assess the uncertainty of these estimates. Because we were unable to calculate CIs for the estimated number of cases not detected by surveillance, we used computer simulation to generate an overall distribution of the number of cases that could plausibly be attributed to chicken consumption; the estimated range was 10,000–105,000 cases annually. This represents a conservative approach to assessing the contribution of chicken consumption to *Campylobacter* infections in Australia. Thus, although we concur that the inclusion of cooked chicken as a risk factor for *Campylobacter* infections was debatable on strictly statistical grounds, we believe that including cooked chicken in our estimates of the PAR for and community incidence of these infections was reasonable.

## References

[1] Gillespie I. Population-attributable risk estimates for *Campylobacter* infection, Australia. Emerg Infect Dis. 2009;15:850–1. 10.3201/eid1505.08155319402998PMC2687014

[2] Evans MR, Ribeiro CD, Salmon RL. Hazards of healthy living: bottled water and salad vegetables as risk factors for *Campylobacter* infection. Emerg Infect Dis. 2003;9:1219–25.1460945510.3201/eid0910.020823PMC3033096

[3] Studahl A, Andersson Y. Risk factors for indigenous *Campylobacter* infection: a Swedish case–control study. Epidemiol Infect. 2000;125:269–75. 10.1017/S095026889900456211117949PMC2869598

[4] Eberhart-Phillips J, Walker N, Garrett N, Bell D, Sinclair D, Rainger W, Campylobacteriosis in New Zealand: results of a case–control study. J Epidemiol Community Health. 1997;51:686–91. 10.1136/jech.51.6.6869519133PMC1060567

[5] Effler P, Ieong MC, Kimura A, Nakata M, Burr R, Cremer E, Sporadic *Campylobacter jejuni* infections in Hawaii: associations with prior antibiotic use and commercially prepared chicken. J Infect Dis. 2001;183:1152–5. 10.1086/31929211237847
